# An Adaptive Coupling of Edge-Based Smoothed FEM and SPH with a Bidirectional Element-Particle Transformation Algorithm for Laser Powder Bed Fusion

**DOI:** 10.3390/ma19112264

**Published:** 2026-05-27

**Authors:** Ming Suo, Ting Long

**Affiliations:** School of Mechanical Engineering, Guangxi University, Nanning 530004, China; yhbm660@163.com

**Keywords:** smoothed particle hydrodynamics (SPH), edge-based smoothed finite element method (ES-FEM), bidirectional adaptive coupling, thermo-fluid-solid coupling, laser powder bed fusion (LPBF)

## Abstract

**Highlights:**

- An adaptive bidirectional ES-FEM-SPH coupling algorithm with element-particle transformation is proposed for thermo-fluid-solid simulation in laser powder bed fusion.
- The algorithm includes a nodal mass normalization scheme, a ghost particle coupling algorithm, and a bidirectional transformation algorithm between finite elements and particles.
- Bidirectional conversion between mesh-free Lagrangian SPH and Lagrangian FEM is realized for the first time with mass conservation.
- The method enables fully coupled simulation of the temperature field, melt flow dynamics, and thermal stress evolution throughout the LPBF process.

**Abstract:**

Laser powder bed fusion (LPBF) poses significant simulation challenges due to its highly nonlinear thermo-fluid-solid coupling. To address this, we propose an adaptive framework coupling the edge-based smoothed finite element method (ES-FEM) and smoothed particle hydrodynamics (SPH) via a bidirectional element-particle transformation algorithm. This integration leverages ES-FEM for modeling solid thermo-mechanical responses and SPH for resolving melt pool dynamics, enabling fully coupled simulation of temperature, fluid flow, and stress within a unified model. The framework comprises three key components: a nodal mass normalization scheme ensuring conservation during transformations, a ghost particle algorithm for solid-fluid heat transfer and interaction, and a bidirectional finite-element-to-particle conversion mechanism. This work represents the first implementation of bidirectional coupling between mesh-free Lagrangian SPH and Lagrangian FEM. The validation against benchmark cases confirms the framework’s accuracy in capturing transient thermal, hydrodynamic, and mechanical behavior. It successfully reproduces key LPBF phenomena, including melt pool morphology, Marangoni flows, and residual stress evolution, demonstrating its suitability for high-fidelity LPBF process simulation. It should be noted that the current ES-FEM-SPH framework has not taken into account the recoil pressure, evaporation, and the interaction between the powder and the molten pool. The powder is regarded as a rigid body. Future work will focus on incorporating these neglected physical factors to further improve the predictive capability of the proposed framework.

## 1. Introduction

Laser Powder Bed Fusion (LPBF), a representative metal additive manufacturing technology, shows significant potential in aerospace [1,2,3] and biomedical engineering [4,5]. However, the process faces critical mechanical challenges: thermal residual stresses cause deformation and cracking [6,7], while unstable melt pool flow triggers defects like porosity and spatter [8,9,10]. Although experimental studies [11,12] provide empirical foundations, they struggle to unravel multi-physics coupling mechanisms and incur high costs. Thus, numerical simulation offers an economical and efficient alternative.

The LPBF process involves multi-field coupling phenomena, including heat transfer and convection, gas–liquid interaction, powder melting and solidification, and solid thermal stress. It spans a broad range of temporal and spatial scales [13,14]. Therefore, the construction of a high-fidelity multi-physics coupling numerical model is of paramount importance. Existing simulation methods can be categorized into three types based on their focus on specific physical fields. These include thermo-mechanical coupling models (typically employing the finite element method (FEM) [15,16,17,18], which neglects melt pool flow), thermo-fluid coupling models (prioritizing melt pool dynamics, treating solids as high-viscosity fluids, and utilizing computational fluid dynamics (CFD) methods such as the finite volume method (FVM) [19,20,21], lattice Boltzmann method (LBM) [22,23], smoothed particle hydrodynamics (SPH) [24,25,26], and particle finite element method (PFEM) [27,28]), and thermo-fluid-solid coupling models. The third category enables the simultaneous solution of melt pool flow and solid stress–strain responses, yet its implementation is highly complex. Most existing studies adopt a one-way coupling strategy. For example, Mukherjee et al. [29] simplified the geometry to calculate residual stresses, while Chen et al. [30] interpolated the CFD temperature field onto FEM grids. However, this approach requires frequent remeshing. Liang et al. [31] introduced a thermo-elasto-plastic constitutive model and developed an accurate mapping algorithm. Some scholars have attempted strong coupling solutions. For example, Wang et al. [32] used the hot optimal transportation meshfree (HOTM) method but omitted the Marangoni force, while Lian et al. [33] proposed a multi-physics material point method (MPH) accounting for Darcy damping and the Marangoni effect. Yang et al. [34,35] unified the solution of fluid and solid domains based on the FVM and considered the influence of solid-state phase transformation on constitutive behavior.

Eulerian methods are well-established for fluid solutions but face challenges in free surface tracking, making it difficult to capture the dramatic surface changes in the melt pool. As a widely used meshless method, SPH offers advantages in free surface tracking, avoiding mesh distortion, and handling multiphase discrete systems. Liu et al. [36] established a surface tension model based on SPH and successfully simulated the morphology of laser melt pools. Afrasiabi et al. [25] employed multi-resolution SPH to enhance the computational efficiency of LPBF. Long et al. [37] developed an SPH model considering recoil pressure and light reflection. Ma et al. [26] constructed a multi-resolution multiphase SPH model integrating ray tracing and wetting effects.

Despite SPH’s achievements in melt pool dynamics, its solid mechanics calculations suffer from low accuracy. In contrast, FEM is more suitable for solid mechanics due to its high accuracy. Thus, there is an urgent need to develop bidirectional transformation and coupling techniques between mesh-based and particle-based methods. Existing SPH-FEM coupling studies [38,39,40,41,42] and element-particle transformation algorithms [43,44,45,46] primarily focus on impact dynamics problems and fluid–structure interaction problems and are mostly limited to one-way transformation from elements to particles. However, the LPBF process involves repeated melting and solidification, necessitating efficient particle-to-mesh transformation. Li et al. [47] proposed a bidirectional MPM-FEM transformation algorithm and applied it to additive manufacturing. However, no research on an adaptive bidirectional element-particle transformation algorithm for coupling FEM and SPH has been reported.

In addition, Long et al. developed the ES-FEM-SPH coupling method for fluid–structure interaction problems [48] and thermo-fluid-solid coupling problems [38]. This method fully utilizes the advantages of ES-FEM in modeling thermo-solid coupling and SPH in modeling thermo-fluid coupling. The smoothed finite element method was proposed by Liu et al. [49] based on the gradient smoothing technique introduced by Chen et al. [50]. Compared with the FEM-SPH coupling algorithm, the ES-FEM-SPH coupling algorithm has unique advantages in modeling thermo-fluid-solid interaction problems. Moreover, no research on an adaptive bidirectional element-particle transformation algorithm for coupling ES-FEM and SPH has been reported in additive manufacturing.

This paper proposes an adaptive bidirectional ES-FEM-SPH coupling algorithm for solving thermo-fluid-solid coupling problems. In this algorithm, ES-FEM handles solid domain calculations with a nodal mass normalization scheme to ensure mass conservation during transformation, while SPH simulates melt pool flow. Heat conduction is solved independently, with interaction realized through a ghost particle coupling algorithm. The transformation criterion is based on temperature analysis and α-shape determination and a simplified method is proposed to implement Darcy damping. The algorithm enables bidirectional transformation while preserving physical quantity conservation. The accuracy of the method is verified through numerical examples and applied to LPBF simulation.

The structure of this paper is as follows. Section 2 outlines the governing equations, the SPH solver, and the edge-based smoothed finite element (ES-FEM) solver. Section 3 details the proposed adaptive bidirectional ES-FEM-SPH coupling algorithm (ABEC), including the nodal mass particleization scheme for ES-FEM, the thermo-fluid-solid coupling algorithm, and the bidirectional element-particle transformation algorithm that enables seamless mutual conversion between finite elements and SPH particles. Section 4 validates the accuracy of the method through numerical examples and presents LPBF simulation results. Section 5 summarizes the full text.

## 2. Numerical Models

The LPBF process encompasses powder spreading, melting of the metal powder and the substrate by a laser heat source, melt pool flow, and solidification cooling. In this work, the powder spreading step is simplified: only a single layer of two-dimensionally arranged metal powder particles with uniform size and spacing is laid. A Gaussian heat source is used to model the laser heat input. The flow behavior of the melt pool is simulated using SPH, while the solid domain is analyzed with the ES-FEM to capture residual stresses.

### 2.1. Governing Equation for Solid Mechanics

In the solid domain, the momentum equation and heat conduction equation under the Lagrangian description can be expressed as

(1) $$\rho^{s}{\ddot{u}}_{i}^{s}=\sigma_{ij,j}+b_{s}^{i},$$

(2) $$\rho^{s}c^{s}{\dot{T}}^{s}=k^{s}T_{,ii}^{s}+Q,$$

where ${\ddot{u}}_{i}^{s}$ is the component of the acceleration vector, $\sigma_{ij}$ denotes the Cauchy stress tensor, $b_{s}^{i}$ represents the body force, $\rho^{s}$, $c^{s}$ and $k^{s}$ denotes the solid density, specific heat capacity, and thermal conductivity coefficient, respectively. Furthermore, $T^{s}$ denotes the absolute temperature, Q signifies the rate of heat generation within the material.

The thermal strain of linear thermoelastic materials affected by temperature is

(3) $$\varepsilon_{ij}^{T}=\alpha_{T}^{s}\left(T^{s}-T_{0}^{s}\right),$$

where $\varepsilon_{ij}^{T}$ denotes the thermal strain, and $\alpha_{T}^{s}$ denotes the coefficient of thermal expansion. The expression for the total strain is given as

(4) $$\varepsilon_{ij}=\varepsilon_{ij}^{e}+\varepsilon_{ij}^{T},$$

where $\varepsilon_{ij}$ is the total strain, $\varepsilon_{ij}^{e}$ is the elastic strain. The Saint Venant–Kirchhoff constitutive [51] model is given as

(5) $$\sigma_{ij}=\lambda \varepsilon_{kk}\delta_{ij}+2\mu \varepsilon_{ij}-\beta^{s}\left(T^{s}-T_{0}^{s}\right)\delta_{ij},$$

where λ, μ are Lame constants, $\delta_{ij}$ is the Kronecker delta. $\beta^{s}$ is the stress temperature modulus, the expression is given as

(6) $$\beta^{s}=\alpha_{T}^{s}\left(3\lambda +2\mu \right).$$

This paper adopts the stress update algorithm and the Jaumann stress rate, which is given as

(7) $${\dot{\sigma }}_{ij}=\sigma_{ij}^{\nabla }+\sigma_{ik}\Omega_{jk}+\sigma_{jk}\Omega_{ik},$$

(8) $$\sigma_{ij}^{\nabla }=\lambda {\dot{\varepsilon }}_{kk}\delta_{ij}+2\mu {\dot{\varepsilon }}_{ij}-\beta^{s}T^{s}\delta_{ij},$$

where ${\dot{\sigma }}_{ij}$ is the Cauchy stress rate, $\sigma_{ij}^{\nabla }$ is the Jaumann stress rate, $\Omega_{ij}$ is the spin tensor.

#### 2.1.1. The Smoothed Finite Element Method

**Figure 1:**
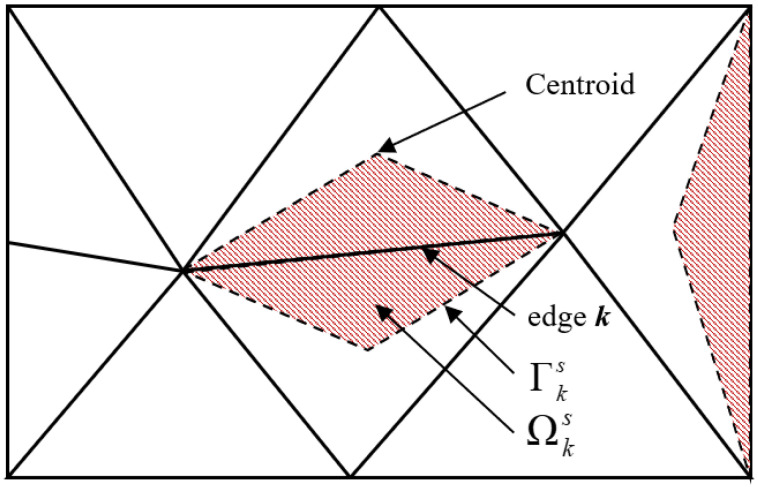
Edge-based smooth domain partitioning for triangular elements.

In the finite element method, physical quantities f(x) are approximated as

(9) $$f(x)=N_{I}f{\left(x\right)}_{I},$$

where $N_{I}$ are the shape functions, $f{\left(x\right)}_{I}$ is the value of the physical quantity at node *I*. In the ES-FEM, the problem domain is divided into a set of no overlap smoothing domains [52,53]. The smooth domain partitioning of the triangular mesh is illustrated in Figure 1. Taking the velocity field as an example, the velocity gradient over the smooth domain is

(10) $${\bar{v}}_{i,j}^{s}=\frac{1}{A_{k}^{s}}\underset{\Omega_{k}^{s}}{\int }\frac{\partial v_{i}^{s}}{\partial x_{j}}d\Omega =\bar{\nabla N_{Ij}}v_{Ii}^{s},$$

(11) $$\bar{\nabla N_{Ij}}=\frac{1}{A_{k}^{s}}\underset{\Gamma_{k}^{s}}{\int }N_{I}n_{j}d\Gamma =(1/A_{k}^{s})\sum_{i=1}^{N_{l}}l_{i}n_{j}N_{I}\left(x_{i}^{G}\right),$$

where $A_{k}^{s}$ is the area of the smoothing domain $\Omega_{k}^{s}$, $\bar{\nabla N_{Ij}}$ is the smoothed derivatives of shape function. Applying the divergence theorem, the smoothed derivatives of shape function are not necessary to compute the derivative of the linear shape function and only require the values of the linear shape functions. Where $n_{j}$ is the unit outward normal vector of the smoothing domain boundary $\Gamma_{k}^{s}$. $N_{l}$ denotes the number of segments of $\Gamma_{k}^{s}$, $l_{i}$ is the length of the boundary, $x_{i}^{G}$ denotes the boundary midpoint (the Gauss integration point), and $N_{I}\left(x_{i}^{G}\right)$ denotes the value of the shape function at the Gauss integration point.

By analogy, the smoothed gradient of temperature can be derived as

(12) $${\bar{T}}_{,j}^{s}=\bar{\nabla N_{Ij}}T_{I}^{s}.$$

By leveraging the smoothed velocity gradient and smoothed temperature gradient, the stress–strain relationship and heat conduction can be efficiently solved.

#### 2.1.2. Discretization of the Solid Domain Governing Equations

Based on the ES-FEM and the weak-form Galerkin variational principle, the following can be derived

(13) $$\underset{\Omega }{\int }\delta \bar{\left(\frac{\partial v_{i}^{s}}{\partial x_{j}}\right)}{\bar{\sigma }}_{ji}d\Omega +\underset{\Omega }{\int }\delta v_{i}^{s}\rho^{s}{\ddot{u}}_{i}^{s}d\Omega =\underset{\Omega }{\int }\delta v_{i}^{s}\rho^{s}b_{i}^{s}d\Omega +\underset{\Gamma_{t}}{\int }\delta v_{i}^{s}{\bar{t}}_{i}{d\Gamma }_{t},$$

where δv is the virtual velocity, ${\bar{\sigma }}_{ji}$ is the Cauchy stress, ${\bar{t}}_{i}$ and $\Gamma_{t}$ denote the surface traction vector and its associated boundary.

(14) $$\begin{array}{c} \underset{\Omega }{\int }\left[\left(\rho^{s}c_{p}^{s}\frac{\partial T^{s}}{\partial t}\right)+\left(\frac{\partial \delta T^{s}}{\partial x_{j}}\right)\left(k^{s}{\bar{T}}_{,j}^{s}\right)\right]d\Omega =\\ \underset{\Gamma_{2}}{\int }\delta T^{s}qd\Gamma +\underset{\Gamma_{3}}{\int }\delta T^{s}h\left(T_{\infty }^{s}-T^{s}\right)d\Gamma , \end{array}$$

where q is the heat flux, h is the heat convection coefficient.

Using the linear shape function and its smooth gradient, Equation (13) can be rewritten as

(15) $$\underset{\Omega }{\int }\bar{\nabla N_{Ij}}{\bar{\sigma }}_{ji}d\Omega +\underset{\Omega }{\int }N_{I}\rho^{s}N_{J}{\ddot{u}}_{i}^{s}d\Omega =\underset{\Omega }{\int }N_{I}\rho^{s}b_{i}^{s}d\Omega +\underset{\Gamma_{t}}{\int }N_{I}{\bar{t}}_{i}{d\Gamma }_{t}.$$

Equation (14) can be rewritten as

(16) $$\begin{array}{c} \underset{\Omega }{\int }\left[\left(\rho^{s}c_{p}^{s}N_{I}N_{J}\frac{\partial T_{J}^{s}}{\partial t}\right)+\bar{\nabla N_{Ij}}\bar{\nabla N_{Jj}}k^{s}T_{J}^{s}\right]d\Omega \\ =\underset{\Gamma_{2}}{\int }N_{I}qd\Gamma +\underset{\Gamma_{3}}{\int }N_{I}h\left(T_{\infty }^{s}-N_{J}T_{J}^{s}\right)d\Gamma . \end{array}$$

The ES-FEM formulation of the momentum equation is

(17) $$M_{I}{\ddot{u}}_{Ii}^{s}+\underset{\Omega }{\int }\bar{\nabla N_{Ij}}{\bar{\sigma }}_{ji}d\Omega =\underset{\Omega }{\int }N_{I}\rho b_{i}d\Omega +\underset{\Gamma_{t}}{\int }N_{I}{\bar{t}}_{i}d\Gamma ,$$

where $M_{I}$ is the lumped mass for the node *I*.

The heat conduction equation of ES-FEM can be written as

(18) $$C_{IJ}{\dot{T}}_{J}^{s}+K_{IJ}T_{J}^{s}=P_{q_{I}}+P_{H_{I}},$$

(19) $$K_{IJ}=\underset{\Omega }{\int }\bar{\nabla N_{Ij}}\bar{\nabla N_{Jj}}k^{s}d\Omega ,$$

(20) $$P_{q_{I}}=\underset{\Gamma_{2}}{\int }qN_{I}d\Gamma ,$$

(21) $$P_{H_{I}}=\underset{\Gamma_{3}}{\int }hT_{\infty }N_{I}d\Gamma ,$$

where $C_{IJ}$ is the lumped mass heat capacity matrix, $K_{IJ}$ is the heat conduction matrix, $P_{q_{I}}$ is heat flux, $P_{H_{I}}$ is heat convection.

### 2.2. Governing Equation for Fluid Dynamics

In this paper, the flow behavior of the molten pool is simulated via the SPH, which is based on the weakly compressible assumption [54,55]. The governing equations of mass, momentum and energy are given as

(22) $$\frac{d\rho }{dt}+\rho \nabla \cdot v=0,$$

(23) $$\rho \frac{dv}{dt}=-\nabla p+\mu \nabla^{2}v+g+F^{b},$$

(24) $$\rho \frac{dT}{dt}=\frac{1}{C_{p}}\nabla \cdot (k\nabla T)+\frac{Q^{b}}{C_{p}},$$

where ρ is the density, v is the velocity, p is the pressure, μ is the dynamic viscosity, g is the gravitational acceleration. T is the temperature, $C_{p}$ is the specific heat capacity, k is thermal conductivity. $F^{b}$ and $Q^{b}$ are external forces and external heat sources. The equation of state (EOS) [56] is used, and it is given as

(25) $$p=c^{2}\left(\rho -\rho_{0}\right),$$

where $\rho_{0}$ is the reference density and c is a numerical speed of sound, its value is typically ten times the maximum particle velocity.

#### 2.2.1. SPH Approximations

The SPH method is a meshless Lagrangian approach [57]. SPH discretizes the computational domain and physical quantities using a set of particles. The gradient of the physical quantity for each particle is approximated based on the weights of neighboring particles. The SPH approximation for $f\left(x_{a}\right)$ and its gradient at position $x_{a}$ can be expressed as

(26) $$f\left(x_{a}\right)=\sum_{b=1}^{N}f\left(x_{b}\right)\frac{m_{b}}{\rho_{b}}W_{ab},$$

(27) $$\nabla f\left(x_{b}\right)=\sum_{b=1}^{N}f\left(x_{b}\right)\frac{m_{b}}{\rho_{b}}\nabla W_{ab},$$

where ab is two interacting particles, W is a kernel function. This paper employs the cubic spline kernel function [58] as the kernel function.

#### 2.2.2. Discretization of the Governing Equations for the Fluid Domain

The equations of mass, momentum and energy conservation are solved by the δ^+^-SPH [59]. The governing equations can be expressed as

(28) $$\frac{d\rho_{a}}{dt}=\rho_{a}\sum_{b}\frac{m_{b}}{\rho_{b}}v_{ab}\cdot \nabla_{a}W_{ab}+0.1hcD_{\rho },$$

(29) $$\rho_{a}\frac{dv_{a}}{dt}=-\sum_{b}\frac{m_{b}}{\rho_{b}}(p_{a}+p_{b})\nabla_{a}W_{ab}+2\hat{\mu }\sum_{b}\frac{m_{b}}{\rho_{b}}\frac{x_{ab}\cdot \nabla_{a}W_{ab}}{{\left|x_{ab}\right|}^{2}}v_{ab}+F^{b},$$

(30) $$\rho_{a}\frac{dT_{a}}{dt}=2\frac{1}{C_{p}}\sum_{b}\frac{m_{b}}{\rho_{b}}\hat{k}(T_{a}-T_{b})\frac{x_{ab}\cdot \nabla_{a}W_{ab}}{{\left|x_{ab}\right|}^{2}}+\frac{Q^{b}}{C_{p}},$$

where $D_{\rho }$ is a numerical diffusive term [60], $\hat{\mu }=\frac{\mu_{a}+\mu_{b}}{2},\;\hat{k}=\frac{2k_{a}k_{b}}{k_{a}+k_{b}},$ $v_{ab}=v_{a}-v_{b}$. $D_{\rho }$ is written as

(31) $$D_{\rho }=\frac{m_{b}}{\rho_{b}}\frac{x_{ab}\cdot \nabla_{a}W_{ab}}{{\left|x_{ab}\right|}^{2}}(2(\rho_{a}-\rho_{b})-\left[\nabla \rho_{a}^{L}+\nabla \rho_{b}^{L}\right]\cdot x_{ab}),$$

(32) $$\nabla \rho_{a}^{L}=\sum_{b}\left(\rho_{a}-\rho_{b}\right)L_{a}\cdot \frac{m_{b}}{\rho_{b}}\nabla_{a}W_{ab},$$

(33) $$L_{a}={\left[\sum_{b}(x_{b}-x_{a})⊗\frac{m_{b}}{\rho_{b}}\nabla_{a}W_{ab}\right]}^{-1},$$

where $L_{a}$ is called the kernel gradient correction (KGC) [61]. The KGC is used to improve the computational accuracy. A particle shifting technique [62] is applied in this paper.

(34) $$F^{b}=F^{s}+F^{bou}$$

(35) $$\delta_{p}=\frac{1}{\Delta x},$$

(36) $$F^{s}=\delta_{p}(\sigma k_{a}\hat{n}+\frac{d\sigma }{dT}(\nabla_{s}T-(\nabla_{s}T\cdot \hat{n})\hat{n})),$$

(37) $$F^{bou}=-\rho_{0}g\alpha_{f}^{T}(T_{a}-T_{0}),$$

where $F^{s}$ is the surface tension, $\delta_{p}$ transforms surface forces into volume forces, Δx is the particle spacing, σ is the surface tension coefficient, $k_{a}$ is the curvature, $\hat{n}$ is the unit normal vector, $\nabla_{s}$ is surface gradient. In this paper, the improved surface tension model [55] is applied. $F^{bou}$ is the boussinesq approximation, $\alpha_{f}^{T}$ is the thermal expansion coefficient of fluid. The SPH model does not take into account the recoil pressure of the molten pool.

### 2.3. Material Description and Heat Source Model

When phase transformation occurs in metallic materials, latent heat of transformation is involved. In this paper, the equivalent specific heat capacity method [63] is employed to approximate the latent heat of transformation. The expression is given as

(38) $$c_{p}=\left\{\begin{array}{c} c_{p}^{s},T\leq T_{s}-\delta T/2\\ \frac{c_{p}^{s}+c_{p}^{f}}{2}+\frac{L}{\delta T},T_{s}-\frac{\delta T}{2}<T\leq T_{s}+\delta T/2,\\ c_{p}^{f},T>T_{s}+\delta T/2 \end{array}\right.$$

where $c_{p}^{s}$ is the specific heat capacity of the material in the solid state, $c_{p}^{f}$ is the specific heat capacity of the material in the liquid state, L is the latent heat of phase transformation, δT is the bandwidth of the solid–liquid transition temperature of the material, $T_{s}$ is melting point of materials.

In this paper, a Gaussian heat source [64] is employed as the laser heat source, with simultaneous consideration of surface convective heat loss [65] and surface radiative heat loss [65]. They are given as

(39) $$Q^{b}=Q^{l}+Q^{c}+Q^{r},$$

(40) $$Q^{l}=\delta_{p}(\alpha \frac{AP_{L}}{\pi R^{2}}e^{\frac{-A\Delta r^{2}}{R^{2}}}),$$

(41) $$Q^{r}=-\delta_{p}\sigma_{B}\varepsilon \left(T_{m}^{4}-T_{\infty }^{4}\right),$$

(42) $$Q^{c}=-\delta_{p}h_{f}(T_{m}-T_{\infty }),$$

where $Q^{l}$ is the Gaussian heat source, $Q^{c}$ is the surface heat convection loss, $Q^{r}$ is the surface heat radiation loss, α is thermal absorption coefficient, A is equal to 2, $P_{L}$ is laser power, R is laser radius, Δr is distance from the center of the laser, $\sigma_{B}$ is Boltzmann constant, ε is radiation coefficient, $T_{\infty }$ is reference temperature, $h_{f}$ is thermal convection coefficient. The external heat source does not take into account the heat loss caused by the evaporation of the molten pool.

## 3. Adaptive Bidirectional ES-FEM-SPH Coupling Algorithm

This paper presents an adaptive bidirectional coupling algorithm that enables reversible transformation between elements and particles. Specifically, when the temperature of the solid domain exceeds the melting point, the corresponding finite element (FE) nodes are automatically discretized into SPH particles for molten pool flow calculations. Conversely, when SPH particles cool and approach solidification (i.e., their temperature falls below the solidus line), their positions and states are mapped to FE nodes and reconstructed into finite elements. This process dynamically updates the geometric boundaries of each computational domain while ensuring conservation and continuity of physical fields.

In the full process simulation of LPBF, FEM governs heat conduction and thermo-mechanical coupling in the solid domain, whereas SPH is used to model molten pool dynamics, mushy zone flow, and splashing particle behavior. The adaptive mutual transformation between the two methods is achieved via a temperature-based conversion criterion and a bidirectional coupling algorithm that maintains the consistency of physical quantities.

The adaptive bidirectional coupling algorithm includes normalization of finite element node mass, a ghost particle coupling method for dealing with heat transfer between ES-FEM and SPH and for fluid–structure interaction problems, and a bidirectional transformation algorithm between finite elements and particles.

### 3.1. Node Mass Normalization Scheme

The bidirectional transformation algorithm proposed in this paper is based on the interconversion between FE nodes and SPH particles. To ensure mass conservation and enable bidirectional node-particle transformation, the mass of each FE node must be equivalently mapped to SPH particles that share an identical mass.

Zhang [66] developed a particle-cell hybrid (PCH) method, which achieves the particleization of node mass by adjusting the size of the node smoothing domain in the node-based particle finite element method (node-based PFEM). Inspired by this work, this paper presents an improved node mass equivalence approach. Without resorting to node integration, the FE integration domain is equivalently partitioned based on the equivalent particle mass. This eliminates reliance on specific integration schemes, thereby enhancing the algorithm’s generality.

**Figure 2:**
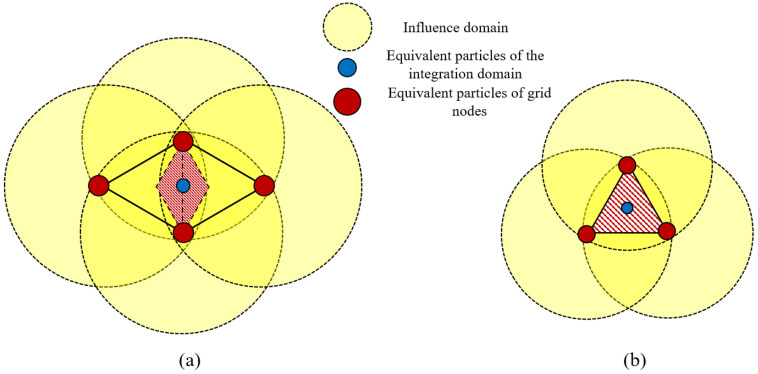
The integration domain and the node mutually influence each other. (**a**) The integration domain and the node mutually influence each other in the ES-FEM. (**b**) The integration domain and the node mutually influence each other in the FEM.

**Figure 3:**
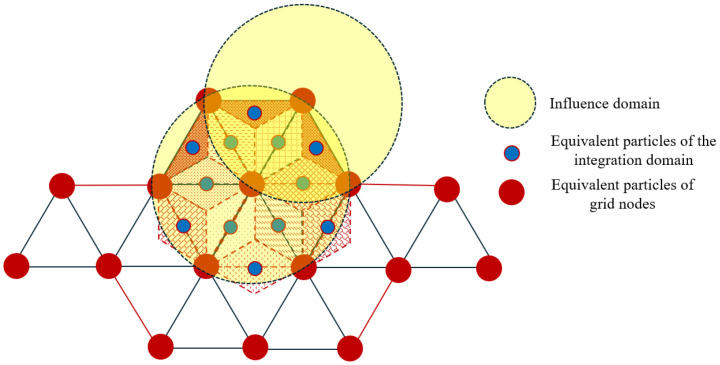
The smooth integral domain of the node in ES-FEM.

Herein, taking the ES-FEM as an illustrative example, the number of nodes $N_{P}$ influenced by the integration domain containing Gaussian integration points is first determined. For internal edges, $N_{P}=4$, for boundary edges, $N_{P}=3$, as shown in Figure 2. To quantitatively characterize the influence degree of the integration domain on each node, this study adopts the particle approximation concept and influence domain principle from the SPH method. Specifically, as shown in Figure 3, the influence domain of each node is defined as the region that encloses its affected integration domains. Based on this definition, a smooth integration volume centered at the node is constructed, which is denoted as ${\tilde{V}}_{\mathrm{node}}$. The expressions are given as

(43) $$x_{G_{j}P_{i}}=x_{G_{j}}-x_{P_{i}},$$

(44) WGP=αd12qGP3−qGP2+23,0<qGP≤1 αd16(2−qGP)3,1<qGP≤2 0,otherwise,

(45) $$q_{GP}=\left|x_{GP}\right|/h_{p},$$

(46) $$\varphi_{G_{j}P_{i}}=W_{G_{j}P_{i}}/\sum_{i=1}^{N_{p}}W_{G_{j}P_{i}},$$

(47) $${\tilde{V}}_{node}=\sum_{j=1}^{N_{G}}\varphi_{G_{j}P_{i}}V_{G_{j}},$$

where $x_{G_{j}}$ is the position of the *j*-th Gaussian point or the centroid of the integration domain (ES-FEM), $x_{P_{i}}$ is the position of the *i*-th node affected by the integral domain. $W_{GP}$ is the cubic spline kernel function [58], which is usually used in the SPH method. The size of the influence domain of the node is controlled by $h_{p}$. In this paper, to balance computational efficiency and discretization accuracy, $h_{p}=\frac{2}{3}\Delta x_{mesh}$, where $\Delta x_{mesh}$ represents the grid spacing. $\varphi_{G_{j}P_{i}}$ is the normalized value of the kernel function, and the value of $\alpha_{d}$ does not affect the value of $\varphi_{G_{j}P_{i}}$ because of the normalization of the kernel function. $V_{G_{j}}$ is the size of the integration domain at the Gaussian points. $N_{G}$ is the number of Gaussian points within the influence domain of the node.

When using the ES-FEM, the ${\tilde{V}}_{\mathrm{node}}$ better captures the integral domain of the node compared to the node-based smoothed PFEM (NS-PFEM).

Similar to Zhang’s [66] approach, we rescale the parameter ${\tilde{V}}_{\mathrm{node}}$ to represent the integration domain size corresponding to an equi-mass particle. The expression is given as

(48) Vpar=mparρpar,

(49) ρpar=ρ0(1+3αsT(Ti−T0)),

(50) $${\tilde{V}}_{node}^{new}=V_{par},$$

where $V_{par}$ is the particle-based integration domain equivalent to the node, $\rho_{par}$ is the density of the equivalent particle. In this paper, we assume that only thermal expansion affects the particle density. In this way, we can obtain the sizes of the modified integral domains. The expressions are given as

(51) $${\tilde{V}}_{node}^{new}=\sum_{j=1}^{N_{G}}\varphi_{G_{j}P_{i}}V_{G_{j}}^{P_{i}},$$

(52) $$\varphi_{G_{j}P_{i}}V_{G_{j}}^{p_{i}}=\frac{V_{par}}{{\tilde{V}}_{node}}\varphi_{G_{j}P_{i}}V_{G_{j}},$$

(53) $$V_{G_{j}}^{new}=\sum_{i=1}^{N_{p}}\varphi_{G_{j}P_{i}}V_{G_{j}}^{P_{i}},$$

where $V_{G_{j}}^{P_{i}}$ is the smooth correction volume of the integration domain $G_{j}$ for node $P_{i}$ after the particleization of node $P_{i}$. $V_{G_{j}}^{new}$ is the size of the corrected integral domain. By adjusting the size of the integration domain, we achieved an equivalent particle approximation of node quality.

In addition, we discuss the discrepancies between the correction of integral domains for boundary points and internal points, a topic not explicitly addressed in Zhang’s work [66]. They are given as

(54) $$\varphi_{G_{j}P_{i}}V_{G_{j}}^{p_{i}}=\left\{\begin{array}{l} \frac{V_{par}}{{\tilde{V}}_{node}}\varphi_{G_{j}P_{i}}V_{G_{j}},P_{i}\;\mathrm{is}\;\mathrm{internal}\;\mathrm{node}\\ \varphi_{G_{j}P_{i}}V_{G_{j}},otherwise \end{array}\right.,$$

(55) $$M_{P_{i}}=\left\{\begin{array}{l} m_{par},\nabla \cdot R_{P_{i}}\geq 1.5\\ m_{I},otherwise \end{array}\right.,$$

where $\nabla \cdot R_{P_{i}}$ is the divergence of the position [67] of the node when it acts as a ghost particle in SPH. All internal nodes are corrected to particles of the same mass. When $P_{i}$ is a boundary point, we calculate the divergence of the position through the ghost particles and SPH particles around the node. The divergence is used to determine whether there are sufficient ghost particles and SPH particles near the node. If there are enough, the boundary node is corrected to be an equal-mass particle; otherwise, the physical quantity change in the node is still calculated in the FEM format. $m_{I}$ is the node mass when the finite element uses a lumped mass matrix.

It should be particularly noted that the integration domain ${\tilde{V}}_{\mathrm{node}}$ of a node only encompasses the range of the local integration region corresponding to the Gaussian integration points directly involved in the gradient calculation of that node. Therefore, there is no need to adopt the linked list traversal strategy that relies on the search for neighboring particles as in the SPH method, which significantly enhances the numerical calculation efficiency.

### 3.2. Ghost Particle Coupling Algorithm

**Figure 4:**
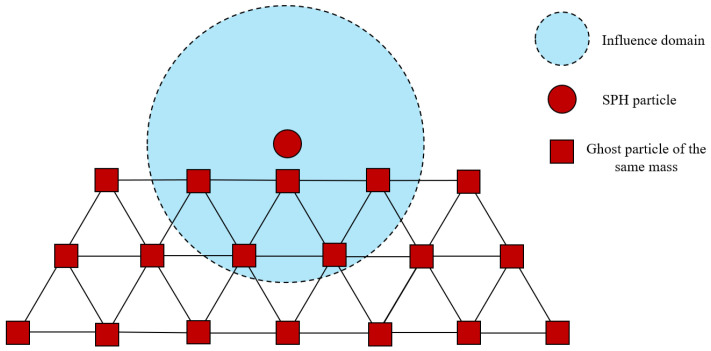
Ghost particles with equal mass are generated at the nodes.

A ghost particle coupling algorithm is developed to handle heat transfer between solid and fluid as well as fluid-solid interactions. As illustrated in Figure 4, by leveraging the node-based equivalent particleization algorithm introduced earlier, both boundary nodes and internal nodes adjacent to the SPH particles can be treated as particles with equal mass to the SPH particles. This eliminates the need for algorithms to generate complex ghost particles within the mesh; instead, the proposed method only requires the generation of ghost particles with uniform mass at the nodes. For the TFSI problem, it is essential to satisfy the kinematic conditions governing fluid–structure interaction, as well as the continuity of the temperature field and the conservation of heat exchange during conjugate heat transfer between the fluid and structure. Consequently, this algorithm incorporates force-related pressure interpolation of ghost particles and temperature field-related heat conduction calculations.

In this paper, the physical quantities of ghost particles are interpolated using the boundary conditions proposed by Adami [68]. They are given as

(56) $$p_{g}=\frac{\sum_{f}p_{f}W_{gf}+(g_{f}-a_{g})\cdot (x_{g}-x_{f})\rho_{f}W_{gf}}{\sum_{f}W_{gf}},$$

where $p_{g}$ is the ghost particle interpolation pressure, $p_{f}$ is the SPH particle pressure, $g_{f}$ is the acceleration caused by the SPH volume force, $a_{g}$ is acceleration of the corresponding node for the ghost particle. $x_{g}$ is the position of the ghost particle, $x_{f}$ is the position of the SPH particle.

(57) $$\rho_{g}=p_{g}/c^{2}+\rho_{0},$$

(58) $$v_{g}=v_{node},$$

where $\rho_{g}$ is the density of the ghost particle, $v_{g}$ is the velocity of the ghost particle, $v_{node}$ is the velocity of the node corresponding to the ghost particle.

Given that the force exerted on solid metals by the molten metal pool is negligible, this paper does not compute the force imposed by the SPH particles on the FEM boundary. Instead, only the ghost particles are generated to prevent non-physical penetration.

**Figure 5:**
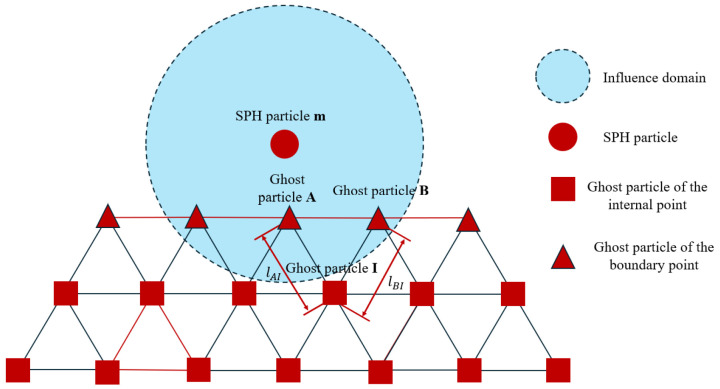
Conjugate heat transfer between ghost particles and SPH particles.

Based on the ghost particle generation approach outlined earlier, this paper proposes a simple and efficient FEM-SPH conjugate heat transfer algorithm. The expressions are given as

(59) $$T_{g}=T_{node},$$

(60) $$\Delta T_{g}=2\frac{1}{\rho_{g}C_{p}}\sum_{f}{\hat{k}}_{gf}\frac{m_{f}}{\rho_{f}}(T_{g}-T_{f})\frac{x_{gf}\cdot \nabla_{g}W_{gf}}{{\left|x_{gf}\right|}^{2}},$$

(61) $${\hat{k}}_{gf}=\frac{2k_{g}k_{f}}{k_{g}+k_{f}},$$

where $T_{g}$, $k_{g}$, $\Delta T_{g}$ are the temperatures of the ghost particles, the thermal conductivity coefficient of the ghost particles, and the temperature change value of the ghost particles. $T_{node}$ is the temperature of the node corresponding to the ghost particle. As shown in Figure 5, by converting the temperature variation in the ghost particle into a heat flux boundary condition, the heat flux boundary variation at boundary point A and the energy equation for particle m under the influence boundary point A of the boundary are derived. The formulas are written as

(62) $$Q_{A}=m_{A}C_{p}\Delta T_{g}^{A}+\sum_{I}^{N_{A}}m_{I}C_{p}\Delta T_{g}^{I}\frac{l_{BI}}{l_{AI}+l_{BI}},$$

(63) $$\left\{\begin{array}{l} \rho_{a}\frac{dT_{a}}{dt}-Q^{b}=2\frac{1}{C_{p}}\sum_{b=1}^{N_{f}+N_{g}}\frac{m_{b}}{\rho_{b}}\hat{k}(T_{a}-T_{b})\frac{x_{ab}\cdot \nabla_{a}W_{ab}}{{\left|x_{ab}\right|}^{2}}\\ \rho_{a}\frac{dv_{a}}{dt}-F^{b}=-\sum_{b=1}^{N_{f}+N_{g}}\frac{m_{b}}{\rho_{b}}(p_{a}+p_{b})\nabla_{a}W_{ab}+\\ \;\;\;\;\;\sum_{b=1}^{N_{f}+N_{g}}2\hat{\mu }\frac{m_{b}}{\rho_{b}}\frac{x_{ab}\cdot \nabla_{a}W_{ab}}{{\left|x_{ab}\right|}^{2}}v_{ab} \end{array}\right.,$$

where $Q_{A}$ is the thermal flux at boundary node A induced by the SPH particle, $N_{A}$ is the number of boundary node A is the nearest boundary node to the internal node. $l_{AI},l_{BI}$ are the distances between node A and node I, the distances between node B and node *I*. $N_{f}$ is the number of the interacting SPH particles, $N_{g}$ is the number of the interacting ghost particles.

Pressure is interpolated onto the ghost particles, thereby enforcing the no-penetration constraint at the FEM boundary and satisfying the kinematic boundary condition; meanwhile, thermal conduction between SPH particles and ghost particles, combined with boundary conversion of the ghost particles’ thermal energy, ensures that the heat flux absorbed by the boundary particles equals the heat flux released by the SPH particles, thus rigorously upholding energy conservation.

### 3.3. Bidirectional Transformation Algorithm Between Finite Elements and Particles

**Figure 6:**
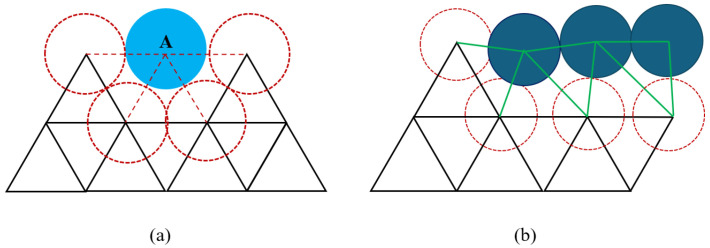
Bidirectional conversion between nodes and particles. (**a**) Node A is converted into an SPH particle. (**b**) Particles undergo deposition and are converted into nodes.

A bidirectional transformation algorithm between finite elements and particles is proposed to achieve a seamless mutual conversion. By introducing a nodal particle-based approximation, the solid–fluid interaction region is approximated as particle-like entities. Consequently, thermally induced phase transitions between the solid and liquid phases—such as deposition and release—are naturally represented through particle insertion and deletion. Meanwhile, the corresponding evolution of the solid boundary is modeled as the dynamic updating of the boundary nodes, as shown in Figure 6. As shown in Figure 6a, when a node is converted into a particle, the elements connected to that node are simultaneously deleted. As shown in Figure 6b, when some particles are converted into element nodes, elements are constructed based on the added nodes using the Delaunay triangulation technique.

The mapping of the physical quantities between nodes and particles is governed by the following expressions

(64) $$\left\{\begin{array}{l} m_{a}=m_{A}\\ v_{a}=v_{A}\\ T_{a}=T_{A}\\ \rho_{a}=\frac{\sum_{f}m_{f}W_{af}}{\frac{m_{f}}{\rho_{f}}W_{af}} \end{array}\right.,$$

(65) mM=mmvM=vmTM=TmρM=ρ0(1+3αsT(TM−T0)),

where $\rho_{a}$ is the current density of the newly generated particle a, $\rho_{M}$ is the density of the new node M when it participates in the finite element calculation. In order to eliminate the interference caused by the SPH particles that are far from the solid domain and on the verge of solidification, this paper employs the Delaunay triangulation technique to conduct meshing for the newly added nodes and removes those triangular elements [27,28] whose circumcircle radius exceeds $\alpha \Delta x_{mesh}$, α=1.3.

Furthermore, inspired by the work of Lian [33], this paper makes a reasonable approximation of the Darcy damping term and explicitly couples its physical effect to the velocity update equation of the particles in the mushy zone, thereby achieving a continuous characterization of the dynamic behavior of the solidification process. This treatment ensures that the SPH particles about to solidify can smoothly and gradually transition from the flow velocity dominated by the liquid phase to the low or near-zero velocity state under solid-phase constraints, effectively suppressing the non-physical velocity jump induced by the local freezing of particles [24,69]. The expressions are given as

(66) $$F_{D}=D_{c}v,$$

(67) $$\alpha_{f}=\left\{\begin{array}{c} \;\;\;0\;\;\;,T_{a}\leq T_{s}\\ (T_{a}-T_{s})/(T_{l}-T_{s}),T_{s}<T_{a}\leq T_{l}\\ \;\;\;1\;\;\;,T_{a}>T_{l} \end{array}\right.,$$

(68) $$D_{c}=C\frac{{\left(1-\alpha_{f}\right)}^{2}}{{\alpha_{f}}^{3}+a},$$

(69) $$\alpha_{D}=\frac{1}{1+\Delta t_{f}D_{c}/\rho_{a}},$$

(70) $$v_{a}=\left\{\begin{array}{l} v_{a},\alpha_{f}=1.0\\ \alpha_{D}v_{a},\alpha_{f}<1.0 \end{array}\right.,$$

where $F_{D}$ is the Darcy damping [70]., C is a coefficient, a is a small constant, $\alpha_{f}$ is the liquid phase fraction, $\Delta t_{f}$ is the single time step size of SPH. $\alpha_{D}$ is the particle velocity correction coefficient for the mushy zone proposed in this paper. As the particle temperature approaches the liquidus infinitely, the corrected velocity is nearly identical to the uncorrected velocity; as the particle temperature approaches the solidus infinitely, the corrected velocity approaches 0 asymptotically.

### 3.4. Numerical Implementation of the Coupling Algorithm

In the SPH, a second-order accurate leapfrog algorithm is employed for explicit time integration. For the ES-FEM, the central difference algorithm is utilized for explicit time integration. The expression governing the time step size constraint is presented as

(71) $$\left\{\begin{array}{l} \Delta t_{CFL}^{f}=0.25\min(\frac{h}{c})\\ \Delta t_{\nu }=0.125\min(\frac{\rho_{a}h^{2}}{\mu_{a}})\\ \Delta t_{T}^{f}=0.1\min(\frac{\rho_{a}C_{p,a}h^{2}}{k_{a}})\\ \Delta t_{\sigma }=0.25\min(\frac{\rho_{a}h^{3}}{2\pi \sigma })\\ \Delta t_{f}=\min(\Delta t_{CFL}^{f},\Delta t_{\nu },\Delta t_{T}^{f},\Delta t_{\sigma }) \end{array}\right.,$$

(72) $$\left\{\begin{array}{l} \Delta t_{CFL}^{s}=0.5\min(\frac{\Delta x_{mesh}}{C_{s}})\\ C_{s}=\sqrt{\frac{E(1-v)}{\rho_{s}(1-2v)(1+v)}}\\ \Delta t_{T}^{s}=0.5\min(\Delta x_{mesh}^{2}\frac{\rho C_{p}}{k_{s}})\\ \Delta t_{s}=\min(\Delta t_{CFL}^{s},\Delta t_{T}^{s}) \end{array}\right.,$$

where $\Delta t_{CFL}^{f}$, $\Delta t_{\nu }$, $\Delta t_{T}^{f}$, $\Delta t_{\sigma }$ are speed of sound constraint, viscous force constraint, heat conduction constraint, surface tension constraint for the SPH. $\Delta t_{CFL}^{s}$, $\Delta t_{T}^{s}$ are stress wave constraints and thermal conduction constraints for ES-FEM. To improve computational efficiency, the mass scaling technique [71] is employed for the stress and strain calculation of the solid domain in LPBF, where the recommended value of the time scaling factor is less than 8000.

**Figure 7:**
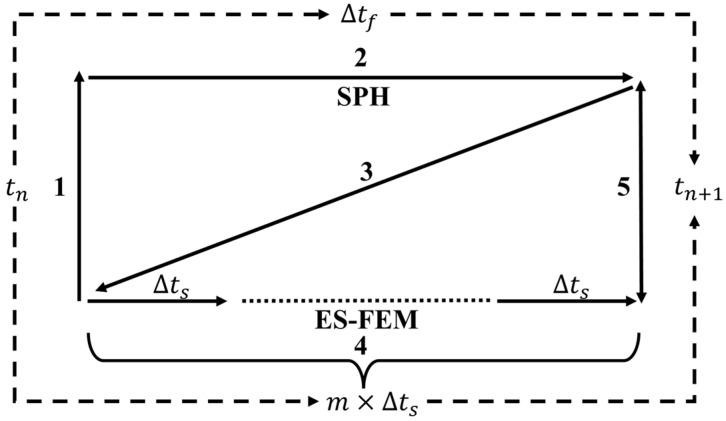
Execution sequence of the adaptive bidirectional ES-FEM-SPH coupling algorithm for a single time step.

The overall numerical implementation of the adaptive bidirectional ES-FEM-SPH coupling algorithm at each time step is illustrated in Figure 7 and summarized as follows:

(1). Dentification of nodes within the influence domain of SPH particles and generation of the ghost particle boundary using node data by Equations (56)–(59) and (63).

(2). The SPH method advances one time step to reach time point $t_{n+1}$, with simultaneous correction of particle velocities in the mushy zone by Equations (66)–(70).

(3). Update the information of the SPH particles, regenerate the ghost particles within the SPH influence domain, and compute the temperature boundary conditions for the ES-FEM by Equations (59)–(62).

(4). Based on the temperature boundary conditions, the ES-FEM advances ***m*** time steps where ***m*** is the least integer ratio of the time step sizes ($\Delta t_{f},\Delta t_{s}$).

(5). Identify the nodes to be converted into particles and the particles to be converted into nodes by Equations (64) and (65), update the boundary mesh, and exclude particles or nodes far from the interface. The entire process is completed within a single time step; subsequently, proceed to the next time step and iterate through the aforementioned steps cyclically.

## 4. Numerical Examples

To validate the accuracy and effectiveness of the proposed adaptive bidirectional ES-FEM-SPH coupling algorithm, five numerical test cases were designed: (1) verifying the method’s precision in transient heat conduction and solid–liquid phase change problems, (2) validating its performance in thermal–solid coupling scenarios, (3) assessing thermal–flow coupling accuracy under molten pool flow conditions, (4) evaluating the feasibility of thermo-fluid-solid coupling under a moving laser heat source, (5) demonstrating the method’s capability to simulate additive manufacturing problems involving molten pool flow effects on the solid domain via a 2D simplified powder-bed LPBF process.

It is worth noting that in conducting the relevant LPBF numerical examples, three simplifying assumptions are adopted in this study, with their limitations clarified as follows: (1) The 2D modeling reduces computational cost but fails to reproduce out-of-plane stresses in 3D scenarios, resulting in deviations from actual results; (2) The thermoelastic assumption focuses on stress concentration induced by thermal stress while ignoring plastic deformation and creep, leading to the overestimation of peak stress; (3) The simplified powder spreading process neglects the interactions between particles and the molten pool. These assumptions are valid for the qualitative analysis of this study, but their limitations still need to be taken into consideration.

### 4.1. Solid–Liquid Phase Change Problem

**Figure 8:**
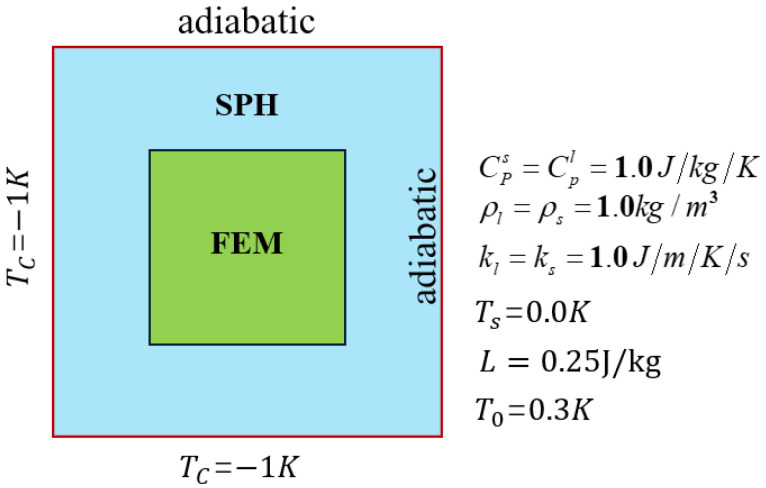
The schematic diagram of 2D phase change problem.

We validate the phase change model within a two-dimensional computational domain [72,73]. It should be noted that this case assumes both the liquid and solid phases remain stationary. The boundary conditions and material parameters are presented in Figure 8. The square domain is filled with liquid at an initial temperature of $T_{0}$, where the left and bottom boundaries are held at a constant temperature $T_{C}$, and the remaining boundaries are thermally insulated. The computational domain has dimensions of 1 m × 1 m. The central region, accounting for approximately one-quarter of the total size, is discretized and solved using the ES-FEM, while the remaining regions are discretized and solved via the SPH method.

**Figure 9:**
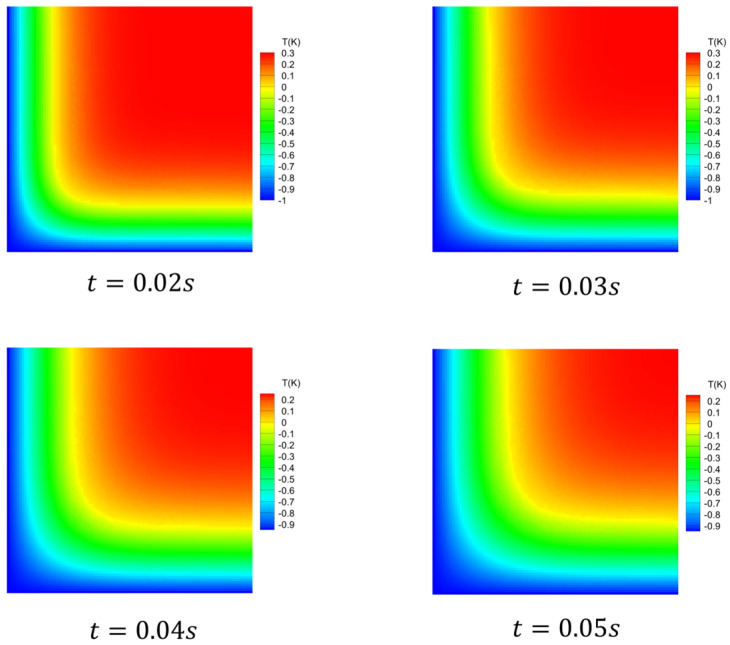
Temperature distributions at different time instants.

The interfaces at four time instants are illustrated in Figure 9. To demonstrate the effect of resolution on numerical error, the temperature distribution and corresponding error along the line y=x at t = 0.02 s were recorded and tabulated in Table 1. From the temperature contour plots at the four time instants, it can be seen that the proposed method can obtain a smooth temperature field. Table 1 results indicate that the numerical error decreases with increasing resolution. Notably, the relatively large error at point (0.3 m, 0.3 m) is attributed to its proximity to the solid–liquid phase interface. It indicates that the proposed adaptive bidirectional ES-FEM-SPH coupling algorithm is effective in modeling the solid–liquid phase change problems.

### 4.2. Thermo-Mechanical Coupling Problem

**Figure 10:**
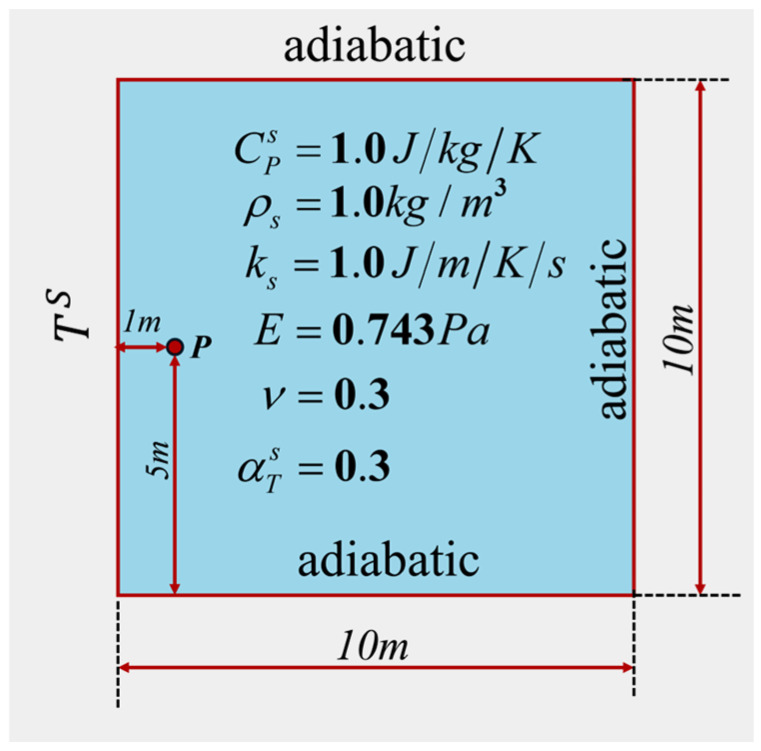
Thermoelastic material parameters and boundary conditions.

The thermo-mechanical coupling capability of the proposed framework is validated against the classical half-space coupled thermoelastic problem. As shown in Figure 10, a square computational domain is modeled under plane strain assumptions. A step temperature increase is imposed on the left boundary, while the top, right, and bottom boundaries are both thermally insulated and mechanically traction-free. The temperature, displacement, and stress components at point P are extracted and quantitatively compared with the analytical solutions derived by Sternberg et al. [74].

The expressions for temperature boundary conditions and dimensionless variables are presented as

(73) $$T^{S}(t)=\Delta T_{H}^{s}H(t)+T_{0}^{s},$$

(74) $$H(t)=\left\{\begin{array}{l} 1,t>0\\ 0,t\leq 0 \end{array}\right.,$$

(75) $$\Delta T_{H}^{s}=0.0001K,$$

(76) $$\begin{array}{l} {\hat{x}}^{s}=\frac{x^{s}}{\chi },\;\hat{t}=C_{1}\frac{t^{s}}{\chi },\;\hat{T}=(T^{s}-T_{0}^{s})/\Delta T_{H}^{s},\;\Delta {\hat{x}}_{i}^{s}=\frac{\lambda +2\mu }{\chi \beta^{s}\Delta T_{H}^{s}}\Delta x_{i}\\ {\hat{\sigma }}_{ij}=\frac{\sigma_{ij}}{\beta^{s}\Delta T_{H}^{s}},\;C_{1}=\sqrt{\frac{\lambda +2\mu }{\rho_{s}}} \end{array}$$

where $T^{S}(t)$ is the temperature boundary condition. ${\hat{x}}^{s}$, $\hat{t}$, $\hat{T}$, $\Delta {\hat{x}}_{i}^{s}$, ${\hat{\sigma }}_{ij}$ are dimensionless position, dimensionless time, dimensionless temperature, dimensionless displacement, dimensionless stress.

**Figure 11:**
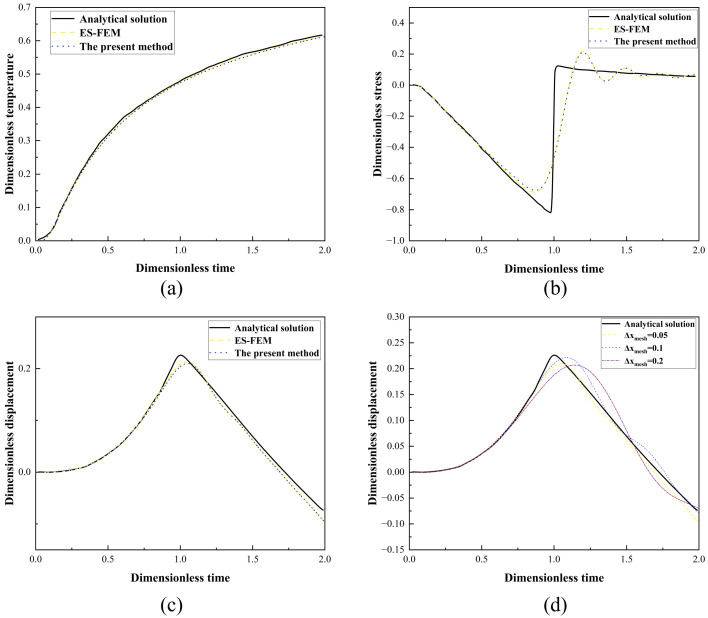
Comparison of dimensionless numbers and analytical solutions. (**a**) Dimensioless temperature. (**b**) Dimensioless stress. (**c**) Dimensioless displacement. (**d**) Grids with different resolutions.

Figure 11 presents a comparison between the numerical results of dimensionless coefficients and the corresponding analytical solution [74]. It is evident that the results derived from the method proposed in this paper exhibit a high degree of consistency with those obtained via the ES-FEM, and both approaches demonstrate excellent agreement with the analytical solutions. This indicates that the proposed method demonstrates effective capability in addressing sequentially coupled thermoelastic problems. As is evident from Figure 11d, the smaller the grid spacing, the closer the results of the present method are to the analytical solution. The stiffness damping is used in this example to suppress stress oscillations and the expression is given as

(77) $$f^{damping}=\beta^{damping}\underset{\Omega }{\int }\bar{\nabla N_{Ij}}\bar{{\dot{\sigma }}_{ji}}d\Omega ,$$

where $\beta^{damping}$ is the damping coefficient, and the recommended value range is 0.005–0.02. ${\bar{\dot{\sigma }}}_{ji}$ is the smoothed stress rate.

**Figure 12:**
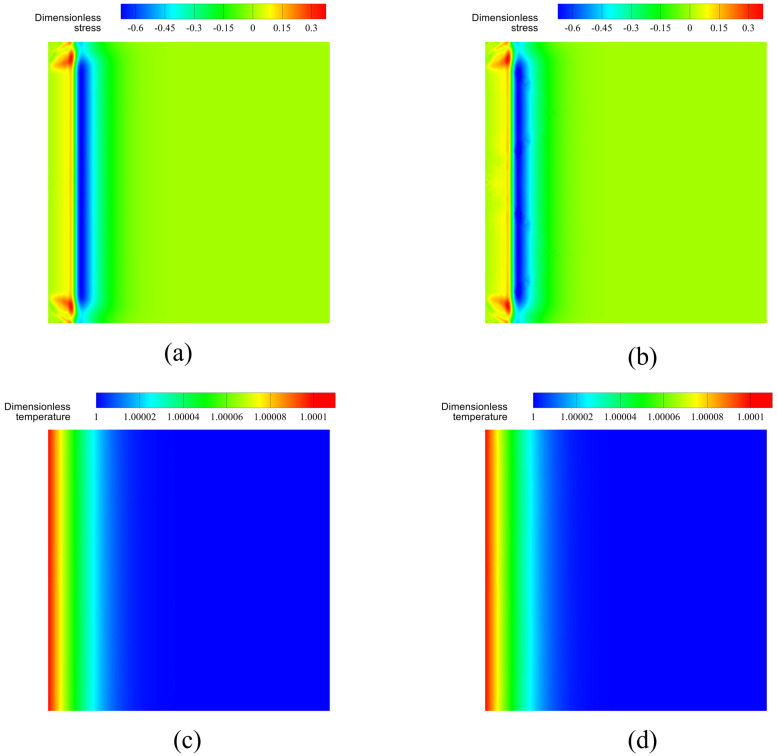
Distribution of dimensionless horizontal stress and temperature at $\hat{t}=1.0$. (**a**) Stress by ES-FEM. (**b**) Stress by this method. (**c**) Temperature by ES-FEM. (**d**) Temperature by this method.

Figure 12 presents the dimensionless temperature and dimensionless horizontal stress at $\hat{t}=1.0$, verifying that the temperature and stress fields solved by ES-FEM are in high agreement with those obtained via the present method.

### 4.3. Thermo-Fluid Coupling Problem in the LPBF Process

In this section, the proposed method is employed to replicate the 304 stainless steel laser-electric welding experiment investigated by He [75], aiming to validate its capability in addressing thermo-fluid coupling problems in metal additive manufacturing.

The computational domain is a rectangular region with dimensions of 1.6 mm × 0.5 mm. A Gaussian laser heat source is applied at the center of the upper boundary of the rectangle and deactivated at t = 3 ms. The parameters required for the simulation are listed in Table 2, with a spatial resolution of 1.0 × 10^−5^. The laser power was set to 1976 W, and the laser radius was 428 μm.

**Figure 13:**
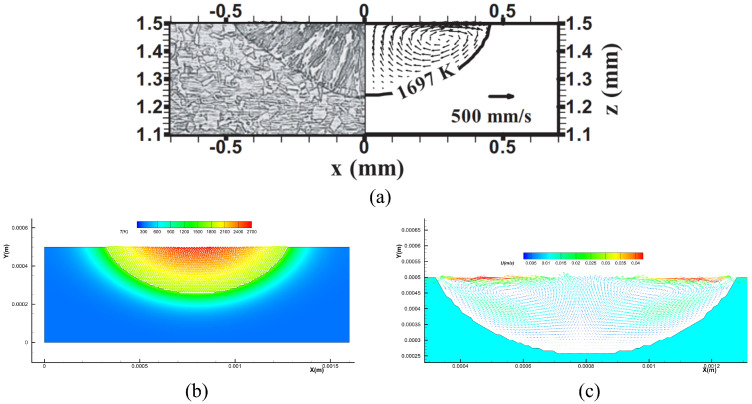
Temperature field and molten pool velocity field results at t = 3 ms. (**a**) Experimental results. (**b**) Temperature field derived from this method. (**c**) Molten pool velocity field derived from this method.

Figure 13 presents a comparative analysis of the velocity fields derived from two distinct approaches. Figure 13a illustrates the velocity distribution obtained via FEM simulations conducted by He [75], and Figure 13b displays the temperature field and its corresponding molten pool contour computed by the present method under identical conditions, and Figure 13c depicts the velocity field within the molten pool solved using the present method.

The overall distribution characteristics of the velocity field generated by the present method exhibit a high degree of consistency with He’s [75] FEM results. Furthermore, the molten pool width and depth derived from the present method are 0.96 mm and 0.25 mm, respectively, which show excellent agreement with the experimental measurements (0.96 mm in width and 0.26 mm in depth). Of particular interest is the observation that near the solid–liquid interface, the particle velocity in the mushy zone is significantly lower than that in the high-temperature molten region, approaching zero—a phenomenon consistent with the physical constraint mechanisms governing solidification processes.

This consistency validation demonstrates that the present method can accurately characterize the thermal-fluid-phase change coupling behavior in the metal molten pool. Furthermore, the introduced mushy zone velocity correction strategy effectively mitigates non-physical discontinuities in the velocity field across the solid–liquid interface, thereby enhancing the numerical stability of the interface transition region.

**Figure 14:**
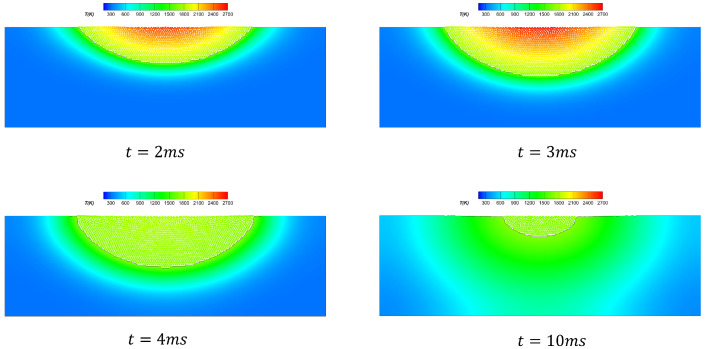
Temperature fields and molten pool contours at different time points during spot welding.

Figure 14 presents the temperature fields and molten pool dimensions at distinct time instants during the spot welding process. During 0–3 ms, the molten pool expands continuously under the laser heat source. After the heat source terminates, the molten pool continues to grow for a brief duration because the metal melt near the heat source center retains a relatively high temperature. At 10 ms, bulk solidification is nearly complete, yet a small residual molten region persists at the former heat-affected zone center.

To quantitatively compare the computational efficiency under the same conditions, additional simulations of the same problem were conducted using the improved SPH method [55] proposed in the previous work. The comparison of computational efficiency is shown in Table 3. The method proposed in this paper has higher computational efficiency than the SPH method at a finer resolution, but there is no significant improvement in computational efficiency. This suggests that we need to further optimize the computational logic of the algorithm in this paper.

Table 4 presents the size of the molten pool obtained by the coupling algorithm at different resolutions and the error compared with the experiments. We can observe that as the resolution increases, the calculated results get closer to the experimental results. Considering both efficiency and accuracy, this paper recommends a resolution of around 5 μm when considering molten pool flow.

**Figure 15:**
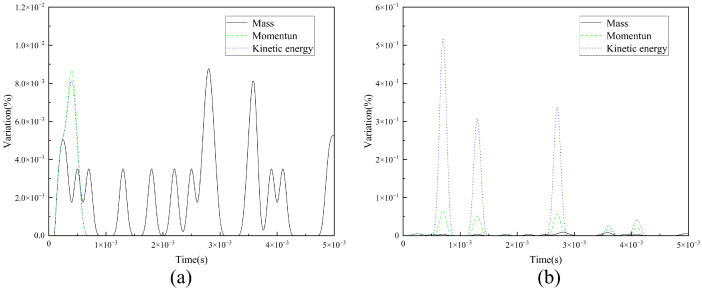
Conservation of mass, momentum and kinetic energy during laser spot welding process. (**a**) The changes in mass, momentum and kinetic energy during transformation with Darcy damping. (**b**) The changes in mass, momentum and kinetic energy during transformation without Darcy damping.

Figure 15 shows the effects of particle-node conversion on mass, momentum and kinetic energy. We can observe that the conversion algorithm has a negligible impact on mass, momentum and kinetic energy, with a magnitude of 10^−5^. Additionally, the Darcy damping approximation proposed in this paper alleviates the momentum and kinetic energy loss caused by the freezing treatment when SPH particles solidify.

It is noteworthy that the two-dimensional spot welding simulation can be treated as a cross-section of three-dimensional spot welding experiments, and the 2D model is capable of accurately reflecting the size of the molten pool. Meanwhile, due to the relatively low intensity of the heat source employed in laser spot welding, the recoil pressure and heat loss induced by the evaporation of the molten pool exert negligible influence on the dimensions of the molten pool.

This case indicates that elements and particles can adaptively convert into one another, enabling the identification and updating of temperature-based solid–liquid boundaries, and the ghost particle coupling algorithm is effective in dealing with the heat transfer between FEM and SPH and fluid–structure interaction problems, the proposed adaptive bidirectional ES-FEM-SPH coupling algorithm can effectively simulate thermo-fluid-solid coupling problems.

### 4.4. Thermo-Fluid-Solid Coupling Problem in the LPBF Process: Excluding Powder Effects

**Figure 16:**
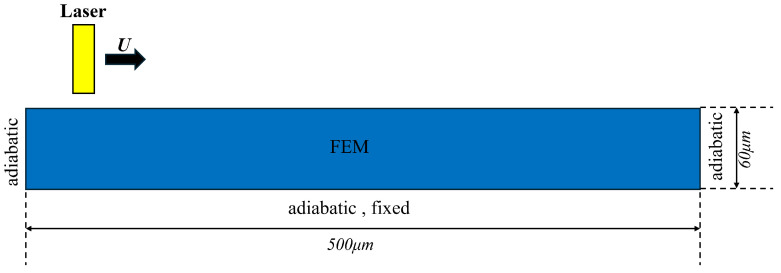
Computational domain for bare plate scanning.

In this section, the proposed method is employed to analyze the laser scanning process on a metal substrate. The material parameters adopted herein are consistent with those presented in Table 2, while the dimensions of the computational domain and boundary conditions are illustrated in Figure 16. The laser power was set to 100 W, and the laser radius was 25 μm, and the laser scanning speed is 2 m/s. The mesh size is set to 2 μm.

**Figure 17:**
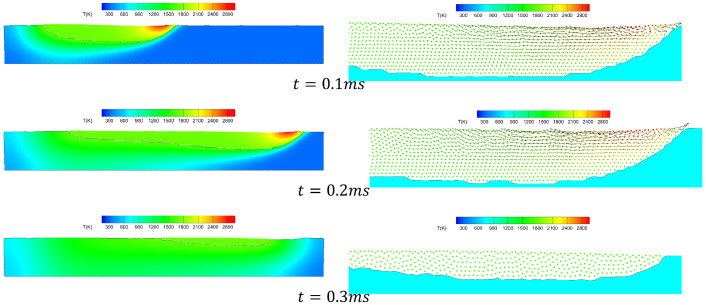
The temperature field and melt pool velocity field at different time periods were scanned by the bare plate.

Figure 17 shows the temperature and velocity fields at selected time instants during laser scanning. During the laser irradiation period (0–0.2 ms), intense localized heating induces a strong surface temperature gradient, driving Marangoni convection: molten metal near the laser spot flows radially outward from the high-temperature center toward cooler peripheral regions, exhibiting relatively high velocities. In contrast, the melt farther from the laser spot remains nearly stagnant and undergoes progressive solidification. After laser shut-off at 0.2 ms, the dominant thermal and hydrodynamic driving force vanishes; consequently, fluid motion is significantly suppressed, and the entire melt pool cools and solidifies progressively under conductive and convective heat loss.

**Figure 18:**
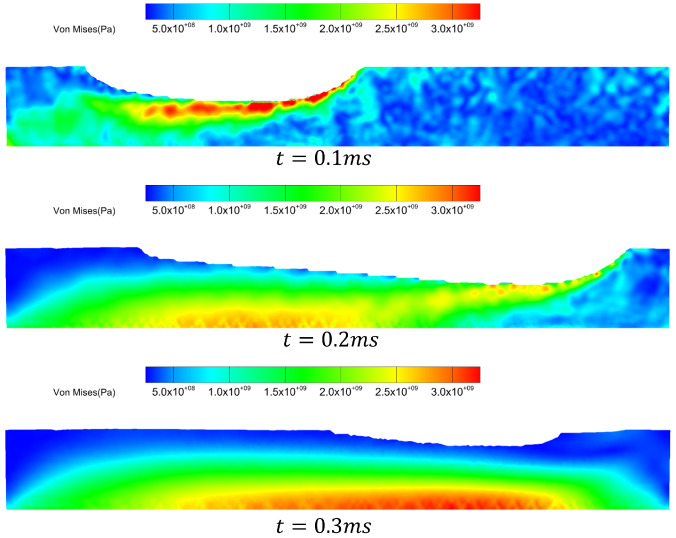
Residual stresses at different time intervals during bare plate scanning.

Figure 18 presents the residual stress distribution at representative time instants during laser scanning. A thermoelastic constitutive model is adopted, and residual stresses are computed solely from thermal expansion mismatch and constrained cooling. As shown, upon laser onset, steep thermal gradients in the vicinity of the heat source induce pronounced stress concentrations near the surface. As scanning proceeds, stress redistribution occurs: the stresses progressively migrate downward and accumulate near the substrate. Following laser shut-off at 0.2 ms, the transient stress concentration at the melt pool boundary dissipates rapidly, while the dominant residual stress field becomes increasingly localized at the substrate base.

It should be emphasized that the current simulation does not account for the effects of recoil pressure and evaporation on the molten pool. Furthermore, under the plane strain thermoelastic assumption, material elastoplasticity and solid-state phase transformations are neglected. These simplifications inevitably introduce discrepancies between the simulation and actual physical conditions. This highlights the need for future work to further refine the melt pool dynamics within this framework and adopt more accurate solid constitutive relations.

### 4.5. Thermo-Fluid-Solid Coupling Problem in the LPBF Process: With Simplified Powder Spreading

**Figure 19:**
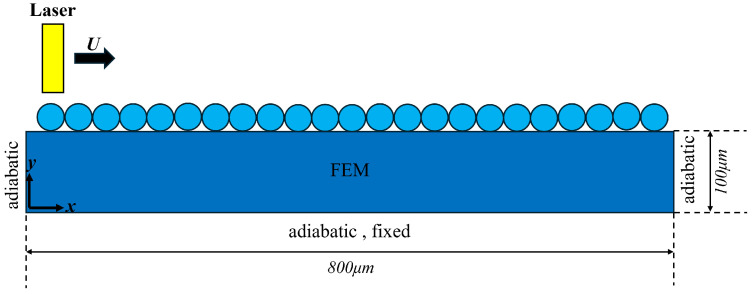
The computational domain of the powder bed.

The computational domain of this case is illustrated in Figure 19. Within this domain, the metal powder is discretized and modeled via the SPH method, with the contact interactions between powder particles neglected. Two monitoring sampling points, *P*_0_ and *P*_1_, are positioned within the computational domain at coordinates (200 μm, 100 μm) and (200 μm, 80 μm), respectively. All material parameters involved are retrieved from Table 2, and the solid mechanics constitutive relation is formulated based on the thermoelastic assumption. The laser power was set to 100 W, the laser radius was 25 μm, and the laser scanning speed was 2 m/s. The radius of the metal powder particles is 32 μm. A uniform spatial discretization scale of 2 μm is adopted, such that both the computational grid spacing and the initial SPH particle spacing are set to 2 μm.

**Figure 20:**
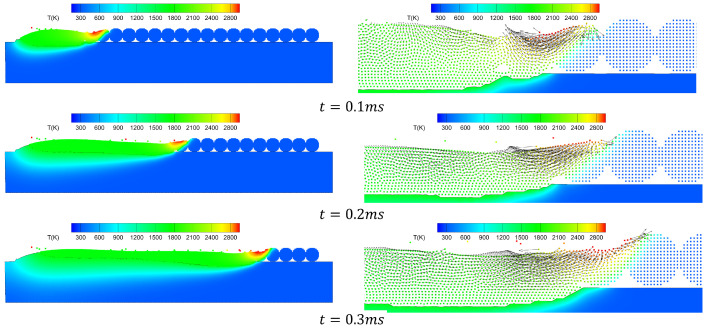
Temperature and melt velocity fields at representative time instants of the powder bed.

Figure 20 illustrates the temperature distribution of the powder bed and the evolution of the velocity field within the melt pool at discrete time instants during the laser scanning process. The results demonstrate that during the laser scanning phase, the Marangoni force governs the melt pool flow, driving the molten metal to migrate from the high-temperature region to the low-temperature region; correspondingly, a fraction of the low-temperature melt region diffuses toward the high-temperature melt region due to the action of the pressure gradient. In the solidification phase following the termination of scanning, the melt pool as a whole tends to be stationary, with the flow essentially ceasing.

**Figure 21:**
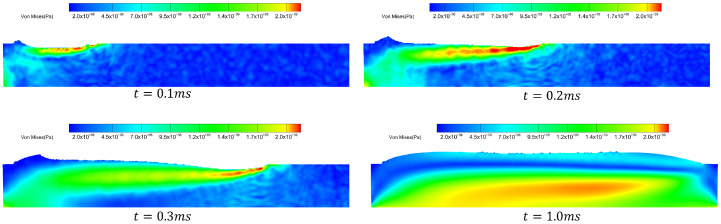
Residual stress fields at representative time instants of the powder bed.

Figure 21 illustrates the residual stress distribution within the powder bed at representative time instants during the laser scanning process. As depicted, upon laser initiation, the steep temperature gradient in the vicinity of the heat source induces pronounced stress concentrations near the solid surface. With the progression of scanning, stress redistribution occurs: the stress gradually migrates downward and accumulates in the region above the substrate. Following laser shut-off, the residual stress in the continuously solidifying metal remains relatively low, while the dominant residual stress field becomes increasingly localized in the area above the substrate.

Similar to the case study in Section 4.4, this simulation does not account for recoil pressure or melt pool evaporation and adopts a thermoelastic constitutive relation for the solid material. On this basis, the powder spreading process is simplified by considering only a single layer of uniformly spaced metal powders with identical particle sizes, while neglecting the interactions between the powder and the molten pool, as well as among the powder particles themselves. In future work, we will incorporate the influence of the powder on the entire physical field by integrating the Discrete Element Method (DEM) based on the SPH framework.

**Figure 22:**
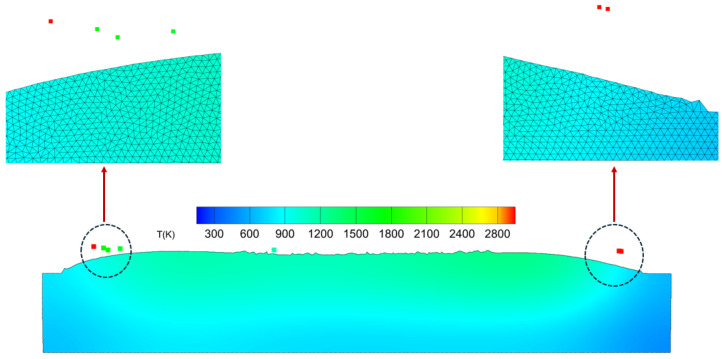
The mesh elements adjacent to SPH particles located far from the primary interface.

**Figure 23:**
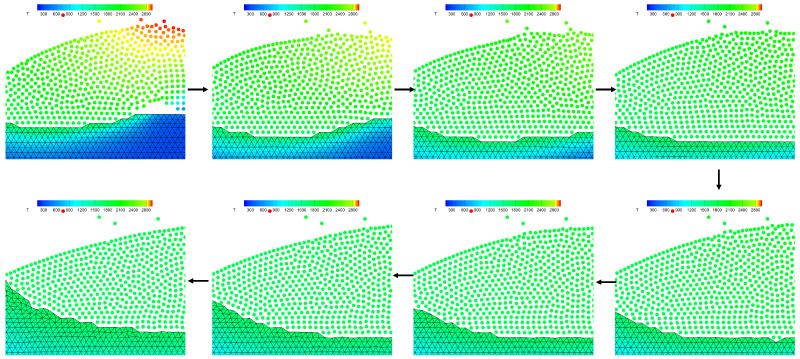
The changes in the grid boundary of some areas.

Figure 22 presents the mesh quality in the vicinity of SPH particles that remain unmeshed after solidification. The mesh elements near the domain boundary exhibit degraded quality (high aspect ratios and skewed angles), whereas interior elements maintain high quality, resulting in a well-defined mesh boundary. Notably, the SPH particles located far from the solidification interface exert negligible influence on mesh. Figure 23 shows the grid update process of a region over eight consecutive output time steps (0.03–0.1 ms). The figure demonstrates that elements and particles can adaptively convert into one another, enabling the identification and updating of temperature-based solid–liquid boundaries.

**Figure 24:**
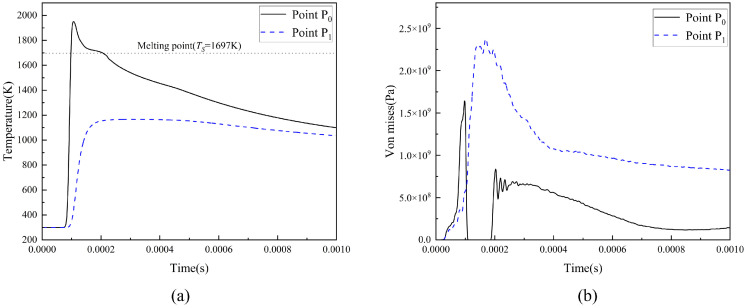
The temperature and von Mises stress at the sampling point. (**a**) Temperature at the sampling point; (**b**) Mises stress at the sampling point.

To intuitively illustrate the evolution of temperature and residual von Mises stress throughout the process, Figure 24 plots the temporal profiles of temperature and von Mises stress at two sampling points (*P*_0_, *P*_1_). Point *P*_1_ is located directly above *P*_0_. At approximately 0.1 ms, *P*_0_ melts and reaches its peak temperature, subsequently, heat conduction causes *P*_0_’s temperature to decrease, while *P*_1_ concurrently attains its maximum temperature which remains below its melting point. After *P*_0_ solidifies, both *P*_0_ and *P*_1_ undergo continuous cooling, and their temperatures gradually converge toward thermal equilibrium. The residual von Mises stress at *P*_0_ increases with rising temperature, drops to near zero upon melting, then rises sharply during solidification and subsequently decreases continuously during cooling. In contrast, the residual von Mises stress at *P*_1_ exhibits a monotonic increase with heating and a gradual decrease with cooling, remaining consistently higher than that at *P*_0_ after *P*_0_’s solidification.

This numerical example further demonstrates that the proposed adaptive bidirectional ES-FEM-SPH coupling algorithm can effectively simulate thermo-fluid-solid coupling problems. Elements and particles can adaptively transform into each other, fully leveraging the advantages of ES-FEM in thermo-mechanical coupling simulation and SPH in thermal-flow coupling simulation, thus achieving coupled temperature-flow-stress modeling. This method holds unique advantages in additive manufacturing simulation.

## 5. Conclusions

In this paper, an adaptive bidirectional ES-FEM-SPH coupling algorithm with a bidirectional element-particle transformation is proposed for simulating thermo-fluid-solid coupling problems. The algorithm is founded on the interconversion between FE nodes and SPH particles. To ensure mass conservation and enable bidirectional node-particle transformation, a nodal mass normalization scheme is introduced, whereby the mass of FE nodes is equivalently mapped to particles of identical mass. A ghost particle coupling method is developed to handle solid-fluid heat transfer and fluid-solid interaction. Moreover, a bidirectional transformation algorithm between finite elements and particles is proposed to achieve seamless mutual conversion.

The accuracy of the proposed method is validated through several numerical cases, including solid–liquid phase change heat transfer, thermo-mechanical coupling, and thermo-fluid coupling, with numerical results compared against analytical solutions or experimental measurements. The method is further applied to two-dimensional LPBF simulations, systematically characterizing the spatiotemporal evolution of the temperature field, melt pool flow dynamics, and residual stress distribution under thermoelastic assumptions.

Compared with conventional multi-physics AM models that rely on segregated solvers and field interpolation, the proposed adaptive bidirectional ES-FEM-SPH coupling algorithm effectively simulates thermo-fluid-solid interactions, allowing elements and particles to adaptively transform into one another. This fully leverages the advantages of ES-FEM in thermo-mechanical coupling and SPH in thermal-flow coupling, thereby achieving coupled temperature-flow-stress modeling. To the best of our knowledge, this work presents the first implementation of a bidirectional conversion scheme between mesh-free Lagrangian SPH and Lagrangian FEM. In summary, the proposed method constitutes an effective computational tool for resolving thermo-fluid-solid coupling in metal additive manufacturing and holds unique advantages in this domain.

Future work will further improve the derivation of 3D algorithms, develop more accurate elastoplastic constitutive models and molten pool dynamics models, and integrate a DEM powder bed to enhance prediction accuracy.

## Figures and Tables

**Table 1:** Temperature value in line *y* = *x* at moment *t* = 0.02 s.

y = x	Analytical Solution	dx = 0.04		dx = 0.02		dx = 0.01	
		Result	Error %	Result	Error %	Result	Error %
0.1	−0.77268	−0.73971	4.26	−0.75673	2.06	−0.76447	1.06
0.2	−0.27256	−0.24765	9.1	−0.25353	6.98	−0.26166	4.00
0.3	0.08567	0.072013	15.94	0.081806	4.52	0.085954	0.326
0.4	0.21854	0.21045	3.70	0.21527	1.50	0.21867	0.060
0.5	0.27685	0.27185	1.81	0.27480	0.740	0.27679	0.02
0.6	0.29491	0.29286	0.700	0.29404	0.295	0.29487	0.014
0.7	0.29912	0.29857	0.184	0.29892	0.067	0.29912	0.0
0.8	0.29988	0.29970	0.06	0.29984	0.013	0.29988	0.0
0.9	0.29999	0.29995	0.013	0.29998	3.33 × 10^−3^	0.29999	0.0

**Table 2:** Physical properties of 304 stainless steel [55,76].

Parameters	Values and Unites	Parameters	Values and Unites
Metal density *ρ*	7200 kg/m^3^	Latent Heat of Fusion L	2.9 × 10^5^ J/kg
Absorption coefficient of heat source α	0.27	Melting Temperature Range Width δT	30.0 K
Darcy damping coefficient *C*	1.0 × 10^6^	Temperature coefficient of surface tension dσ/dT	−4.3 × 10^−4^ N/(m·K)
Solid state thermal conductivity k_S_	19.3 W/(m·K)	Coefficient of surface tension	1.6 N/m
Liquid state thermal conductivity k_f_	209.3 W/(m·K)	Solid state specific heat $C_{P}^{S}$	711.6 kJ/(kg·K)
Temperatura solidi T_S_	1697.0 K	Specific heat of liquid state $C_{P}^{f}$	837.2 kJ/(kg·K)
Reference temperature $T_{\infty }$ (K)	300.0 K	Liquidus T_l_	1727.0 K
Dynamic Viscosity of Solids μs	5.0	Dynamic viscosity of liquids μ_f_	0.1
Coefficient of thermal expansion for solids $\alpha_{T}^{S}$	1.5 × 10^−5^	Coefficient of thermal expansion for liquids $\alpha_{T}^{f}$	2.0 × 10^−5^
Young’s modulus of elasticity *E*	200-0.09(*T*-273) GPa	Poisson’s ratio ν	0.3

**Table 3:** Comparison of computational efficiency for thermo-fluid problems.

Δx (μm)	Δt (s)	The SPH Method	The Proposed Method	Efficiency
20	1.0 × 10^−7^	578 s	566 s	102%
10	1.0 × 10^−7^	2157 s	1615 s	133%
6.667	5.0 × 10^−8^	10,661 s	6142 s	173%

**Table 4:** The molten pool size and error of laser spot welding at different resolutions.

Δx (μm)	Width (mm)	Error %	Depth (mm)	Error %
20	0.941	1.98	0.242	6.92
10	0.956	0.417	0.248	4.62
6.667	0.957	0.313	0.255	1.92

## Data Availability

The original contributions presented in this study are included in the article. Further inquiries can be directed to the corresponding author.
